# Obstacles to successful treatment of hepatitis C in uninsured patients from a minority population

**DOI:** 10.1186/s12967-018-1555-y

**Published:** 2018-06-28

**Authors:** Alexandra DeBose-Scarlett, Raymond Balise, Deukwoo Kwon, Susan Vadaparampil, Steven Xi Chen, Eugene R. Schiff, Gladys Patricia Ayala, Emmanuel Thomas

**Affiliations:** 1 University of Miami School of Medicine, 1600 NW 10th Ave #1140, Miami, FL 33136 USA; 2 H Lee Moffitt Cancer Center and Research Institute, 12902 USF Magnolia Drive, Tampa, FL 33612 USA; 3 Schiff Center for Liver Diseases, 1500 NW 12th Ave #1101, Miami, FL 33136 USA; 4 Jackson Health System, 1005, 1611 NW 12th Ave, Miami, FL 33136 USA; 5 Sylvester Comprehensive Cancer Center, 1550 NW 10th Ave., Papanicolaou Bldg, PAP 514, Miami, FL 33136 USA; 6 Schiff Center for Liver Diseases, 1550 NW 10th Ave., Papanicolaou Bldg., PAP 514, Miami, FL 33136-1015 USA

**Keywords:** Hepatitis C virus, Sustained virologic response, Direct acting antivirals, Uninsured, Minority

## Abstract

**Background:**

Hepatitis C virus (HCV) treatment regimens (DAAs) are well tolerated, efficacious but costly. Their high cost and restricted availability, raises concerns about the outcome of treatment in uninsured patients. This study investigated sustained virologic response (SVR) outcomes in a predominately uninsured patient population and completion of four steps along the HCV treatment cascade.

**Methods:**

A retrospective chart review was conducted to characterize the patient population and analyze covariates to determine association with insurance status, attainment of SVR and progression through the HCV treatment cascade.

**Results:**

Out of a total of 216 patients, 154 (71%) were uninsured. Approximately 50% of patients (109 of 216 patients) were male and 57% were Hispanic (123 of 216 patients). Sex, race, ethnicity, treatment compliance, and rates of complications were not associated with insurance status. Insured patients were older (median 60 years vs 57 years, p-value < 0.001) and had higher rates of cirrhosis: 32 out of 62 patients (52%) vs 48 out of 154 patients (31%) (p-value = 0.005). Insured patients were tested for SVR at similar rates as uninsured patients: 84% (52 of 62 patients) vs 81% (125 of 154 patients), respectively. Of those tested for SVR, the cure rate for insured patients was 98% (51 out of 52 patients) compared to 97% (121 out of 125 patients) in the uninsured. Out of those who completed treatment, 177 of 189 (94%) were tested for attainment of SVR. Compliance rates were significantly different between tested and untested patients: 88% (156 of 177 patients) vs 0% (0 of 12 patients), respectively (p-value < 0.001). However, insurance status, race ethnicity, cirrhosis, and complications were not associated with being tested for SVR.

**Conclusions:**

These results demonstrate that insured and uninsured patients with chronic HCV infection, with access to patient assistance programs, can be treated and have comparable clinical outcomes. In addition, testing for SVR remains an important obstacle in completion of the HCV treatment cascade. Nevertheless, patient assistance programs remove a significant barrier for treatment access in real-world HCV infected populations.

**Electronic supplementary material:**

The online version of this article (10.1186/s12967-018-1555-y) contains supplementary material, which is available to authorized users.

## Background

Hepatitis C virus (HCV) is a significant global health problem [1, 2]. An estimated 130–180 million individuals are currently infected [3], with 3–4 million new infections each year [3]. An estimated 2.7–3.9 million people in the United States currently live with HCV and 15,000 die each year due to HCV disease and resultant hepatic complications [4]. However, these numbers are likely an underestimation of the true disease burden because the highest risk groups are often under-represented in general population studies [5].

Approximately 85% of infections progress to chronicity [3]. Chronic infection can lead to cirrhosis, hepatocellular carcinoma (HCC) [2] and liver failure [6]. Most people are unaware of their infection [7], putting them at increased risk of spreading the disease and more likely to suffer from HCV-related morbidity and mortality [3]. Expanded screening guidelines [8] have increased the number of cases detected [9]. The identification of occult cases not only allows these patients to receive treatment, but decreases the risk of HCV transmission [3].

The introduction of direct acting antiviral medications (DAAs) [2] for the treatment of HCV with fewer side effects [10], and the recommendation that all chronically infected HCV patients be treated [8], has had a significant impact [11]. These medications have sustained virologic response (SVR) rates as high as 97% in clinical trials, compared to approximately 50% with interferon-based regimens [12]. SVR is associated with decreased morbidity and mortality, and improved quality of life [13]. Despite these tangible benefits, insufficient research has been done on real-world treatment outcomes in uninsured HCV infected patients treated with DAAs, and even less in indigent patient populations [14, 15]. Furthermore, while barriers to treatment initiation have been elucidated [16], obstacles to successful treatment completion remain to be fully characterized [17].

Since these treatment regimens are expensive, the role of insurance coverage in HCV treatment is important. Lack of health insurance is associated with decreased pursuit and uptake of HCV treatment, with cost being a driving factor [18]. Having insurance coverage is associated with increased linkage to care [19], faster approval times for treatment [20], and subsequent retention in care [19]. Interestingly, once treatment with protease inhibitors is initiated, uninsured patients have comparable SVR rates to insured patients [15, 21]. Importantly, these studies did not report completion of specific steps within the HCV treatment cascade. Since guidelines now recommend the treatment of nearly all chronically infected patients [8], analysis of treatment outcomes in previously ineligible patients is tantamount to maximizing cure and subsequent eradication of HCV. Accordingly, this study investigates SVR rates and completion of steps in the HCV treatment cascade in a predominately uninsured patient population with access to patient assistance programs.

## Methods

### Study cohort

216 chronic HCV mono-infected patients were treated at Jackson Memorial Hospital since 2014. All patients who were eligible for testing for SVR 12 weeks post treatment completion before August 1, 2017 were included in this retrospective electronic health record (EHR) review. To be eligible for treatment, patients needed to be referred by a primary care provider and assessed for financial assistance eligibility. Individuals needed to be illicit drug and alcohol free for 6 months in non-cirrhotic patient or 3 months for cirrhotic patients. Treatment was selected and initiated based on American Association for the Study of Liver Diseases (AASLD) Guidelines [8], taking into account genotype, cirrhosis status and pre-treatment viral load. Patients who ultimately took their first dose of medication (as documented in the EHR) were included. This study was approved by the Institutional Review Board at the University of Miami and Jackson Health System’s Office of Research and Grants.

### Study outcomes

The primary variables collected were insurance status and the results of a post treatment viral load test 12 or more weeks after cessation of therapy. SVR was achieved if the patient was negative for detectable virus at this time. Additionally, age at treatment initiation, sex, race, ethnicity, genotype, pre-treatment viral load and cirrhosis status were collected. We also assessed whether the patient was compliant with appointments and whether complications were reported. All of these covariates were analyzed as distinct clinical variables.

Age at first treatment with a DAA regimen was documented at treatment initiation and recorded in years. Sex, race, and ethnicity were collected and defined per criteria of the EHR. All patients categorized as Asian, other, or other/white were combined into a single Asian/other race category. Patients who did not list an ethnicity category (n = 4) were classified as non-Hispanic. An additional variable combining race and ethnicity was created in which the non-Hispanic patients were assigned to their designated race categories, such as non-Hispanic black and non-Hispanic white. Non-compliance was defined as missing multiple clinic appointments or laboratory assessments, or being documented in the EHR as a “non-compliant patient”. All other patients were designated as compliant. Complications were defined as any treatment related complication that the patient reported and was recorded in the patient EHR, including headache, fatigue and difficulty understanding instructions. Treatment completion was assessed by patient report and documented in the EHR. The presence of cirrhosis was defined as the patient being documented as having the diagnosis of cirrhosis based on clinical or radiologic criteria.

### Statistical analysis

All statistical analyses were conducted using SAS 9.4. Chi square tests were used for categorical variables. For variables in which expected cell frequencies were less than 5, Fisher Exact Tests were conducted. Continuous variables that were not well described by a normal distribution were tested using Wilcoxon rank-sum tests. Univariable and multivariable logistic regression utilizing all predictor variables was performed to determine associations between potential predictor variables and insurance status and between potential predictors and being tested for SVR. 95% confidence intervals for odds ratios were calculated. Significance was determined at a two-sided alpha level of 0.05 for all tests.

## Results

### HCV treatment in insured and uninsured patients

Initially, we analyzed our data for differences in clinical covariates and treatment outcomes between insured and uninsured patients (Table 1). 216 patients were included in this study and their corresponding data was used for analysis. The mean age of treated patients was approximately 56 years; the youngest was 25 and the oldest was 77. The median age was 58 and the interquartile range was 52–62 years. Males and females were almost equal in number (109 and 107, respectively). The distribution of race, as recorded in the EHR, was 153 whites, 59 blacks, 2 Asians, 1 other and 1 other/white. The documented ethnicity distribution was 123 Hispanics and 89 Non-Hispanics, with four patients lacking an ethnicity designation (of which two were insured and two were uninsured, p-value 0.6990). Most patients were compliant with appointments and laboratory assessments and approximately half experienced some type of complication or side effect of treatment. Pre-treatment viral load ranged from 840 to 166,211,511 IU/mL, with a mean of 4,247,796 and median of 1,655,329 (interquartile range was 434,953–4,676,597). The overall HCV Genotype (GT) distribution was 81% GT1, 9% Type GT2, 7% Type GT3, six patients with GT4, and one patient with a mixed GT1 and GT2 infection. The majority of patients were non-cirrhotic, completed treatment and attained SVR.

**Table 1:** Descriptive statistics on data from insured and uninsured patients

	Total (%)	Insured (%)	Uninsured (%)
N (216)	216 (100%)	62 (29%)	154 (71%)
Age at treatment, years [mean (sd)] (p-value < *0.0001*)	56 (9.3)^a^	58.9 (8.89)^a^	54.2 (9.16)^a^
Sex (p-value 0.3227)			
Male	109 (50%)	28 (45%)	81 (53%)
Female	107 (50%)	34 (55%)	73 (47%)
Race (p-value 0.6111)			
White	153 (71%)	41 (66%)	112 (73%)
Black	59 (27%)	20 (32%)	39 (25%)
Asian/other	4 (2%)	1 (2%)	3 (2%)
Ethnicity (p-value 0.9261)			
Hispanic	123 (57%)	35 (56%)	88 (57%)
Non-Hispanic	93 (43%)	27 (44%)	66 (43%)
Race/ethnicity (p-value 0.1072)			
Hispanic	123 (57%)	35 (56%)	88 (57%)
Non-Hispanic white	38 (18%)	6 (10%)	32 (21%)
Non-Hispanic black	52 (24%)	20 (32%)	32 (21%)
Asian/other	3 (1%)	1 (2%)	2 (1%)
Compliance (p-value 0.1975)			
Yes	165 (76%)	51 (82%)	114 (74%)
No	51 (24%)	11 (18%)	40 (26%)
Complications (p-value 0.2129)			
Yes	111 (51%)	36 (58%)	75 (49%)
No	105 (49%)	26 (42%)	79 (51%)
Pre-treatment viral load, IU/mL [median (range)] (p-value 0.5957)	1,655,329 (840–166,211,511)^b^	1,806,502 (840–166,211,511)^b^	1,527,253 (3,888–> 69,000,000)^b^
Completed treatment^c^ (p-value 0.4261)			
Yes	189 (88%)	56 (90%)	133 (86%)
No	27 (13%)	6 (10%)	21 (14%)
SVR status (p-value 0.8874)			
SVR	172 (80%)	51 (82%)	121 (79%)
No SVR	5 (2%)	1 (2%)	4 (3%)
Not tested for SVR	39 (18%)	10 (16%)	29 (19%)
Genotype (p-value 0.5619)			
1	174 (81%)	52 (84%)	122 (79%)
2	20 (9%)	5 (8%)	15 (10%)
3	15 (7%)	3 (5%)	12 (8%)
4	6 (3%)	1 (2%)	5 (3%)
Mixed	1 (0.46%)	1 (2%)	0 (0%)
Cirrhosis (p-value *0.0049*)			
Yes	80 (37%)	32 (52%)	48 (31%)
No	136 (63%)	30 (48%)	106 (69%)

Italicized p-values are significant

^a^ Mean (standard deviation)

^b^ Median (range)

^c^ Completed treatment per guidelines (based on patient report)

71% of patients in this cohort (n = 154) had no health insurance (Table 1). Insured patients were older (p-value < 0.0001) with a median age of 60 (interquartile range was 56–65) compared to 57 (interquartile range was 50–61) in the uninsured. Sex, race and ethnicity were not significantly associated with insurance status. In addition, compliance was not associated with insurance status. The development of complications or side effects was also not associated with insurance status. The average pre-treatment viral load in the insured was 5,973,784 IU/mL with a median of 1,806,502 (interquartile range 535,360–3,793,470) and it was not statistically different from the uninsured that had a mean of 3,552,918 and median of 1,527,253 (interquartile range 388,600–4,762,484). HCV Genotype distribution was also not associated with insurance status. Similar proportions of insured patients completed treatment as uninsured patients (90% vs 86%, respectively, p-value 0.4261). However, cirrhosis was associated with insurance status with significantly more cirrhotics being insured (40% vs 22%, respectively, p-value 0.0049). Univariable logistic regression revealed similar results as described above, however in multivariable logistic regression, only age and cirrhosis remained significantly associated with insurance status (Table 2). Overall, there were relatively few differences between our insured and uninsured patients.

**Table 2:** Univariable and multivariable logistic regression for association with insurance status

	Univariable logistic regression		Multivariable logistic regression	
	Odds ratio (95% CI)	p-value	Adjusted odds ratio (95% CI)	p-value
Age at treatment, years	0.934 (0.897, 0.974)	*0.0013*	0.942 (0.901, 0.986)	*0.0096*
Sex				
Female vs male (ref)	0.742 (0.411, 1.341)	0.3234	0.890 (0.463, 1.713)	0.7279
Race				
Non-white vs white (ref)	0.732 (0.388, 1.381)	0.3354	–	–
Black vs white (ref)	0.714 (0.374, 1.363)	0.3071	–	–
Asian/other vs white (ref)	1.098 (0.111, 10.858)	0.9361	–	–
Ethnicity				
Hispanic vs Non-Hispanic	1.029 (0.567, 1.865)	0.9260	–	–
Race/ethnicity				
Hispanic vs Non-Hispanic white (ref)	0.471 (0.181, 1.226)	0.1231	0.444 (0.158, 1.246)	0.1231
Non-Hispanic black vs Non-Hispanic white (ref)	0.300 (0.107, 0.845)	*0.0227*	0.291 (0.093, 0.910)	*0.0338*
Asian/other vs Non-Hispanic white (ref)	0.375 (0.029, 4.822)	0.4516	0.333 (0.011, 10.046)	0.5268
Compliance				
No vs yes (ref)	1.626 (0.772, 3.424)	0.2004	1.544 (0.546, 4.367)	0.4127
Complications				
Yes vs no (ref)	0.686 (0.378, 1.243)	0.2140	0.601 (0.309, 1.169)	0.1335
Pre-treatment viral load, IU/mL		0.2738		0.1780
1,000,000 unit increase	0.986 (0.962, 1.011)		0.981 (0.955, 1.009)	
Completed treatment^a^				
No vs yes (ref)	1.473 (0.564, 3.846)	0.4285	2.316 (0.404, 13.283)	0.3459
Tested for SVR				
No vs yes (ref)	1.206 (0.549, 2.653)	0.6408	0.356 (0.068, 1.871)	0.2223
Genotype				
Non-1 vs 1	1.364 (0.625, 2.977)	0.4360	1.208 (0.497, 2.937)	0.6770
2 vs 1	1.279 (0.442, 3.701)	0.6503	–	–
3 vs 1	1.705 (0.462, 6.294)	0.4234	–	–
4 vs 1	2.131 (0.243, 18.692)	0.4946	–	–
Mixed vs 1	NE	NE	–	–
Cirrhosis				
Yes vs no (ref)	0.425 (0.232,0.776)	*0.0054*	0.438 (0.226, 0.847)	*0.0142*

Italicized p-values are significant

Multivariable logistic regression analysis included all variables. *CI* confidence interval

^a^ Completed treatment per guidelines (based on patient report)

### Analysis of the HCV treatment cascade

**Fig. 1:**
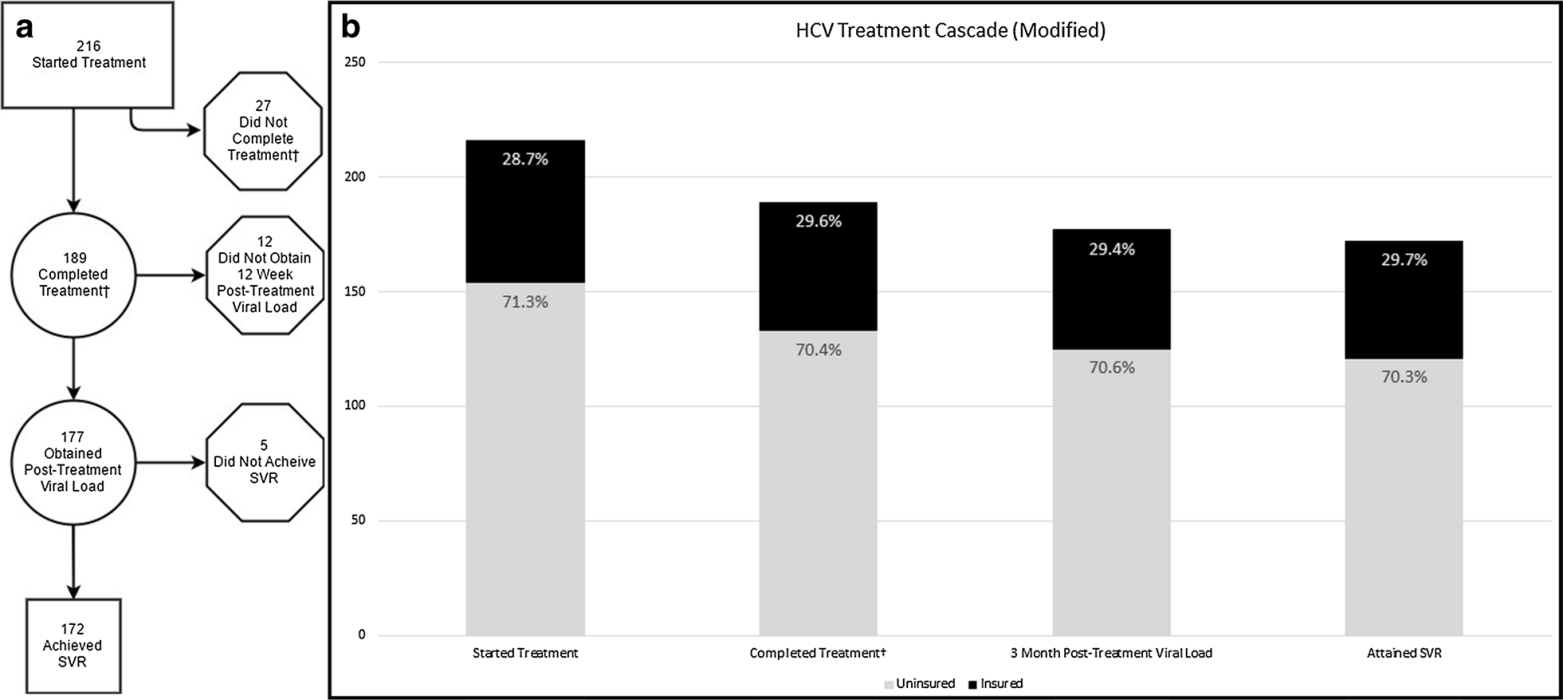
Modified HCV treatment cascade. **a** Absolute counts of patients progressing through the cascade. **b** Percentage of insured and uninsured patients at each step. ^†^Completed treatment per guidelines (based on patient report)

We next assessed the transition of our cohort though the HCV treatment cascade [22] based on insurance status. The HCV care cascade describes the progression of patients from diagnosis of infection to cure [22]. However, the HCV treatment cascade does not include screening, diagnosis, and linkage to care. In order to elucidate clinically relevant transition points in the HCV treatment cascade, we only focused on steps involved specifically in HCV treatment and two steps were added: treatment completion and 12-week post-treatment viral load testing. Figure 1 presents a schematic of the patient numbers as they move through the treatment cascade and it includes data from our complete cohort of 216 patients that initiated HCV treatment. As found in the previous analysis, there were no significant differences between the insured and uninsured patients as they progressed through the HCV treatment cascade. As expected, patient attrition occurred as individuals moved through the treatment cascade.

**Fig. 2:**
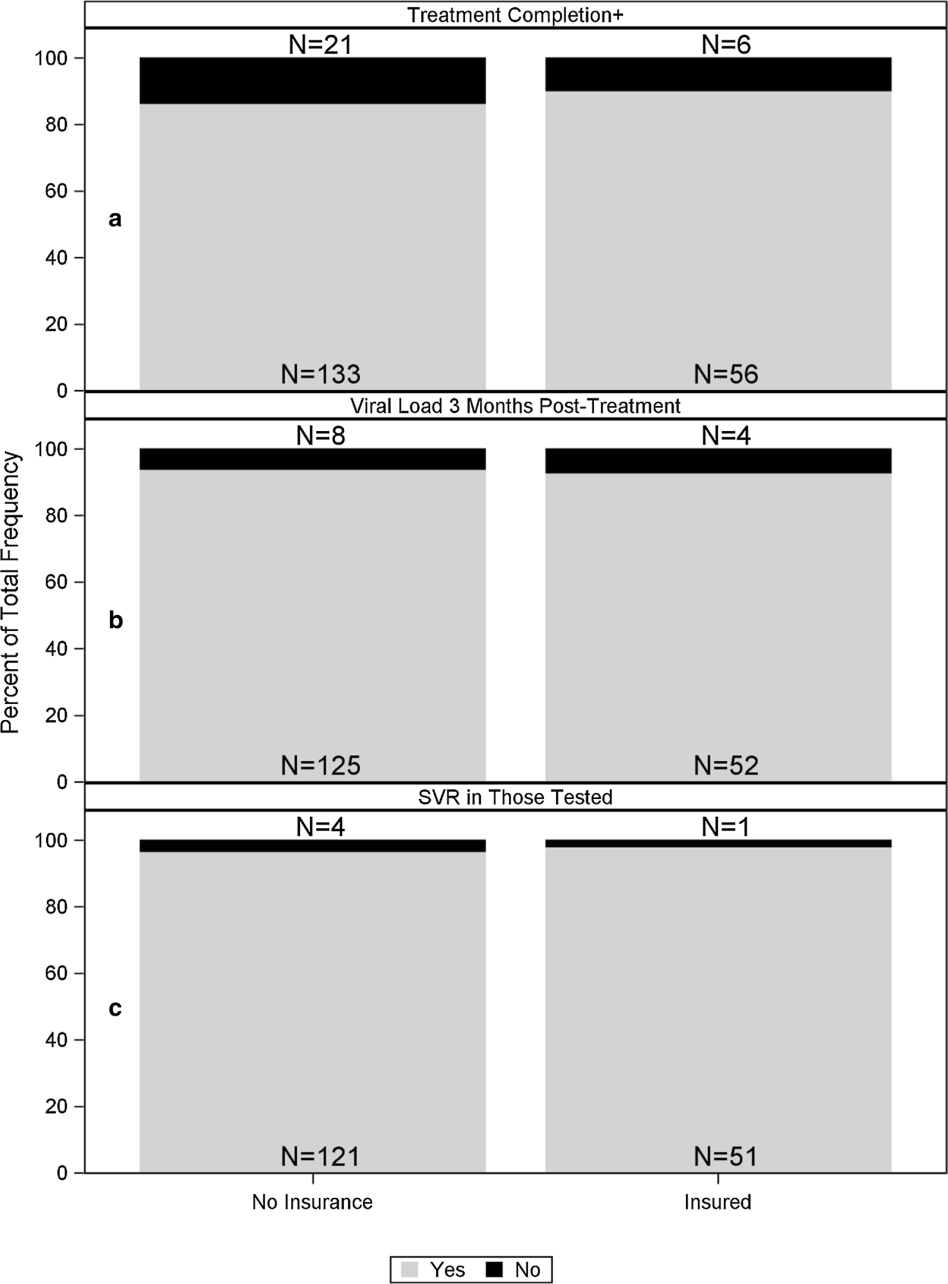
Attainment of specific milestones in the HCV treatment cascade by insured and uninsured patients. **a** In patients who initiated treatment (N = 216). **b** In patients who completed treatment (N = 189). **c** In patients who obtained a 12-week post treatment viral load (N = 177). ^†^Completed treatment per guidelines (based on patient report)

Of the 216 patients who initiated treatment, 87.5% of patients (n = 189) completed treatment (90% of the insured and 86% of the uninsured, p-value 0.4261). Within these 189 patients, 177 (94%) obtained a viral load 12 weeks after treatment completion (93% of the insured and 94% of the uninsured, p-value 0.7516). Within the population that obtained a viral load at 12 weeks post-treatment (177 patients), 172 patients (97%) had undetectable HCV RNA levels achieving SVR (98% of the insured and 97% of the uninsured, p-value 1.0000) (Fig. 2). Interestingly, we found a large unexpected occurrence of patient dropout at the stages of completing treatment and being tested for SVR 12 of more weeks after treatment completion (Fig. 1). Neither of these parameters are typically described as phases of the HCV care cascade [22].

### Sub-group analysis of cirrhotics within the HCV treatment cascade

Interestingly, in those tested for SVR, cirrhotics attained similar rates of cure as non-cirrhotics (95 and 98% respectively, p-value 0.3630). Likewise, cirrhotics and non-cirrhotics had comparable rates of treatment completion (85% vs 89%, respectively, p-value 0.3942) and comparable rates of obtaining a 12-week post treatment viral load to assess the attainment SVR (97% vs 92%, respectively, p-value 0.2169) (Additional file 1: Figure S1).

### Analysis of treatment completion

The largest drop-off of patients along the HCV treatment cascade occurred between treatment initiation and treatment completion. 27 patients (12.5% of the cohort who began treatment) failed to reach the next step of the HCV treatment cascade. These patients were younger (p-value 0.0081), with a median age of 54 years (interquartile range 41–59) compared to a median age of 58 for those who completed treatment (interquartile range 53–62). A greater proportion of females completed treatment than males (93% vs 82%, respectively, p-value 0.0087). Lastly, patients who completed treatment were more compliant (p-value < 0.0001) (Table 3). Race, ethnicity, complications, insurance status, pre-treatment viral load, genotype and cirrhosis were not associated with treatment completion in univariable or multivariable analysis (Additional file 2: Table S1).

**Table 3:** Descriptive statistics on data from patients who completed and did not complete treatment

	Total (%)	Completed^a^ (%)	Did not complete^a^ (%)
N (216)	216 (100%)	189 (87.5%)	27 (12.5%)
Age at treatment, years [mean (sd)] (p-value *0.0081*)	56 (9.3)^b^	56 (8.7)^b^	50 (11.4)^b^
Sex (p-value *0.0087*)			
Male	109 (50%)	89 (82%)	20 (18%)
Female	107 (50%)	100 (93%)	7 (7%)
Race (p-value 1.0000)			
White	153 (71%)	133 (87%)	20 (13%)
Black	59 (27%)	52 (88%)	7 (12%)
Asian/other	4 (2%)	4 (100%)	0 (0%)
Ethnicity (p-value 0.1608)			
Hispanic	123 (57%)	111 (90%)	12 (10%)
Non-Hispanic	93 (43%)	78 (84%)	15 (16%)
Race/ethnicity (p-value 0.2801)			
Hispanic	123 (57%)	111 (90%)	12 (10%)
Non-Hispanic white	38 (18%)	30 (79%)	8 (21%)
Non-Hispanic black	52 (24%)	45 (87%)	7 (13%)
Asian/other	3 (1%)	3 (100%)	0 (0%)
Compliance (p-value < *0.0001*)			
Yes	165 (76%)	156 (95%)	9 (5%)
No	51 (24%)	33 (65%)	18 (35%)
Complications (p-value 0.4402)			
Yes	111 (51%)	99 (89%)	12 (11%)
No	105 (49%)	90 (86%)	15 (14%)
Insurance (p-value 0.4261)			
Yes	62 (29%)	56 (90%)	6 (10%)
No	154 (71%)	133 (86%)	21 (14%)
Pre-treatment viral load, IU/mL [median (range)] (p-value 0.6122)	1,655,329 (840–166,211,511)^c^	1,675,814 (840–166,211,511)^c^	969,891 (12,710–15,600,000)^c^
Genotype (p-value 0.4598)			
1	174 (81%)	153 (88%)	21 (12%)
2	20 (9%)	18 (90%)	2 (10%)
3	15 (7%)	11 (5%)	4 (27%)
4	6 (3%)	6 (100%)	0 (0%)
Mixed	1 (0.46%)	1 (100%)	0 (0%)
Cirrhosis (p-value 0.3942)			
Yes	80 (37%)	68 (85%)	12 (15%)
No	136 (63%)	121 (89%)	15 (11%)

Italicized p-values are significant

^a^ Completed treatment per guidelines (based on patient report)

^b^ Mean (standard deviation)

^c^ Median (range)

### Analysis of 12-week post-treatment viral load adherence

Since treatment completion has been studied in other populations [23] and is influenced by a myriad of clinical factors not necessarily related to insurance coverage, we next analyzed covariates that correlate with adherence for SVR testing (Table 4). Although 189 patients completed treatment, only 177 (82% of the cohort, 94% of those who completed treatment) were assessed for SVR attainment 12 weeks after treatment completion. There were no statistically significant differences in age between those tested and those who were not (p-value 0.8764). Neither sex, race, ethnicity nor insurance status were associated with being tested for SVR. However, as expected, compliant patients were significantly more likely to be tested for SVR than non-compliant patients (p-value < 0.0001). Furthermore, every compliant patient who completed treatment was ultimately tested for SVR, while approximately one-third of non-compliant patients who completed treatment failed to obtain a 12-week post treatment viral load. Importantly, pre-treatment viral load did not differ between patients tested and untested for SVR: median 1,675,814 (interquartile range 330,073–4,736,831) vs 1,172,716 (interquartile range 470,264–4,408,714). Tested and untested patients had similar complication rates, genotype distribution and cirrhosis rates. Univariable logistic regression yielded similar results; however, the compliance odds ratio and corresponding p-value were not estimable because, as stated previously, there were no compliant patients who completed treatment but did not obtain a 12-week post treatment viral load. Analogous results were obtained in multivariable logistic regression (Additional file 3: Table S2).

**Table 4:** Descriptive statistics on data from patients who were and were not tested for SVR

	Total (%)	Tested for SVR (%)	Not tested for SVR (%)
N (189)	189 (100%)	177 (94%)	12 (6%)
Age at treatment, years [mean (sd)] (p-value 0.8764)	56 (8.7)^a^	56 (8.7)^a^	56 (8.9)^a^
Sex (p-value 0.8347)			
Male	89 (47%)	83 (93%)	6 (7%)
Female	100 (53%)	94 (94%)	6 (6%)
Race (p-value 1.0000)			
White	133 (70%)	124 (93%)	9 (7%)
Black	52 (28%)	49 (94%)	3 (6%)
Asian/other	4 (2%)	4 (100%)	0 (0%)
Ethnicity (p-value 0.5565)			
Hispanic	111 (59%)	105 (95%)	6 (5%)
Non-Hispanic	78 (41%)	72 (92%)	6 (7%)
Race/ethnicity (p-value 0.3681)			
Hispanic	111 (59%)	105 (95%)	6 (5%)
Non-Hispanic white	30 (16%)	26 (87%)	4 (13%)
Non-Hispanic black	45 (24%)	43 (96%)	2 (4%)
Asian/other	3 (2%)	3 (100%)	0 (0%)
Insurance (p-value 0.7516)			
Yes	56 (30%)	52 (93%)	4 (7%)
No	133 (70%)	125 (94%)	8 (6%)
Compliance (p-value < *0.0001*)			
Yes	156 (83%)	156 (100%)	0 (0%)
No	33 (17%)	21 (64%)	12 (36%)
Complications (p-value 0.8645)			
Yes	99 (52%)	93 (94%)	6 (6%)
No	90 (48%)	84 (93%)	6 (7%)
Pre-treatment viral load, IU/mL [median (range)] (p-value 0.6178)	1,675,814 (840–166,211,511)^b^	1,675,814 (840–166,211,511)^b^	1,542,867 (95,425–19,635,228)^b^
Genotype (p-value 0.1868)			
1	153 (81%)	144 (94%)	9 (6%)
2	18 (9%)	18 (100%)	0 (0%)
3	11 (7%)	9 (82%)	2 (18%)
4	6 (3%)	5 (83%)	1 (17%)
Mixed	1 (0.46%)	1 (100%)	0 (0%)
Cirrhosis (p-value 0.2169)			
Yes	68 (36%)	66 (97%)	2 (3%)
No	121 (64%)	111 (92%)	10 (8%)

Italicized p-value is significant

^a^ Mean (standard deviation)

^b^ Median (range)

Of the 216 patients who started treatment, 88% completed treatment per guidelines, based on patient self-report (90% of the insured and 86% of the uninsured, p-value 0.4261). Of those who completed treatment, 94% were tested for SVR, or 93% of the insured and 94% of the uninsured. The SVR rate in those tested was 97% (98% in the insured and 97% in the uninsured) (Figs. 1 and 2). Overall, in our study cohort, uninsured patients have similar treatment outcomes and high cure rates as insured patients. However, patients were lost to follow-up as they progressed through the HCV treatment cascade and this was not correlated with insurance status.

## Discussion

In this study, we have investigated HCV treatment outcomes in uninsured and insured patients, at a large safety-net hospital in Miami, Florida. Additionally, compliance and complication rates were investigated. Overall, we found similar clinical outcomes between the two groups. Specifically, our study demonstrates comparable SVR rates (97%) to those reported in clinical trials (95% and higher) in a real-world population of uninsured HCV infected patients. These results undermine the hypothesis that uninsured patients will be less likely to comply with the steps of HCV treatment cascade and thus, less likely to achieve SVR. Our study directly addresses a key provider-level barrier to care for these patients and strengthens the recommendation that all HCV infected patients should be treated for this deadly virus infection [8].

Unfortunately, new DAA regimens can cost more than $5000 per week for the duration of treatment that can be as long as 24 weeks [24]. Lack of insurance is associated with a decreased likelihood of being offered or receiving treatment for HCV infection [25]. Low treatment uptake, defined as initiating treatment after being prescribed DAAs, also remains a barrier to treating HCV effectively [26]. Although some patients have insurance and some may qualify for patient assistance programs to help defray the cost of the medications, the overall cost of care may remain a barrier for some patients [27], and that would include repeated testing for HCV RNA viral load. In addition to the high cost of DAA regimens, traditional barriers to care are augmented for HCV patients [24]. Medication copayments do not take into account physician visit copayments, the cost of traveling to the physician’s office, laboratory testing and the cost of lost wages [24]. For example, the cost of HCV RNA testing for treatment monitoring can cost patients as much as $60 or more, out of pocket. A hepatic function panel may cost an additional $10 to $20 [24]. Thus, some patients labelled as “non-compliant” may simply lack the financial resources necessary to adhere to treatment guidelines and document attainment of SVR. This study reveals an often-overlooked impact of socioeconomic status on the achievement of treatment milestones using highly potent, all oral antiviral therapy for HCV.

In the study cohort, insured patients were older and more likely to be cirrhotic. This may reflect eligibility requirements for Medicare that includes individuals of 65 years or older or having a permanent disability [28]. Cirrhosis can enable a patient to receive social security benefits on the grounds that it is considered a permanent disability [29]. This social security designation can then qualify a patient to be eligible for Medicare benefits [28]. Despite these differences, SVR was achieved in 95% of cirrhotics and 98% of non-cirrhotics who completed all steps of the HCV treatment cascade.

In contrast, the HCV care cascade [22] provides a framework to consider when public policy initiatives are implemented to address the HCV epidemic. However, the traditional HCV care cascade lacks the resolution to identify additional barriers to achieving SVR that are not commonly considered. Approximately 50% of patients are lost between the final two steps of the care cascade (i.e. prescribing HCV treatment and achieving SVR) [22]. Part of this decrease was previously attributed to the poor efficacy of interferon-based treatment regimens; however, this effect is negated when considering the routine use of highly efficacious interferon-sparing DAA regimens. In our cohort, only treated with DAAs, there were still a significant number of patients who were prescribed and initiated treatment, but did not ultimately follow through to the end of the HCV care cascade, that includes achieving a documented cure. Two additional clinically relevant transition points were added to the HCV care cascade, treatment completion and testing for 12-week post-treatment viral load, to facilitate the analysis of additional factors associated with successful progression through the HCV treatment cascade. These two additional steps highlight under-appreciated barriers for patients and clinicians to overcome.

With respect to these additional barriers, of the variables investigated, only compliance remained a significant predictor of failure to complete the HCV treatment cascade. Specifically, if a patient missed appointments or laboratory measures consistently during treatment, this patient continues to be unlikely to complete the HCV treatment cascade. Importantly, insurance status was not associated with completing treatment or being tested for attainment of SVR.

This study is limited by its observational and retrospective design. Patients were not randomized into groups to minimize the effects of unmeasured factors. In addition, clinical markers of liver function were not collected. Furthermore, information on prior HCV treatment and the specific DAA regimen utilized were not analyzed due to the heterogeneity in our cohort arising from patient self-report and lack of information in the EHR. However, all covariates were assessed by chart review to reduce recall bias and the treating physician was included in the data analysis. Finally, since a major outcome of the study was the minimal effect of insurance status on the HCV treatment cascade in patients with access to assistance programs, these limitations are unlikely to influence this conclusion.

A significant strength of this study is its characterization of the real-world experience treating an indigent patient population. DAAs have expanded eligibility for treatment to encompass nearly every patient with HCV infection, extending treatment options to both the insured and uninsured. Additionally, recently expanded screening practices are identifying more patients that could benefit from treatment, but who are also uninsured and lack access to regular medical care. The attainment of SVR in the uninsured may have a disproportionately large impact on HCV transmission and related morbidity since this population may have the highest HCV prevalence [30] and are more likely to transmit HCV [30]. Specifically targeting this uninsured population could therefore dramatically reduce the public health impact of this disease.

This study also highlights an additional barrier in the HCV treatment cascade that includes being tested for SVR. In this cohort, 12.5% of patients who completed treatment did not return for a 12-week post treatment viral load. Patients who have been treated, but in which SVR has not been assessed, pose a unique risk. They may assume that they are cured when they are not and engage in risky behaviors, unknowingly spreading the disease to others. While DAAs are highly effective treatments for HCV, improvements in treating viral hepatitis are still being pursued [31]. However, documentation of SVR and subsequent patient education remain an essential and underappreciated step in treating chronically infected patients, particularly those who are socioeconomically disadvantaged.

## Conclusions

Overall, this study demonstrates that uninsured patients with access to patient assistance programs have similar HCV treatment outcomes as insured patients. Curing the uninsured is likely to have a marked impact on HCV prevalence and transmission. Appropriate treatment and assessment of SVR in HCV infected, uninsured individuals will contribute to the realization of HCV eradication.

## Additional files

**Additional file 1: Figure S1.** Attainment of specific milestones in the HCV treatment cascade of cirrhotic and non-cirrhotic patients. **A**. In patients who initiated treatment (N = 216). **B**. In patients who completed treatment (N = 189). **C.** In patients who obtained a 12-week post treatment viral load (N = 177). † Completed treatment per guidelines (based on patient report).

**Additional file 2: Table S1.** Univariable and multivariable logistic regression for association with treatment completion.

**Additional file 3: Table S2.** Univariable and multivariable logistic regression for association with being tested for SVR.

## Abbreviations

AASLD: American Association for the Study of Liver Diseases
DAA: direct acting antiviral
EHR: electronic health record
GT: genotype
HCC: hepatocellular carcinoma
HCV: hepatitis C virus
RNA: ribonucleic acid
SVR: sustained virologic response
