# Supplementary figures and images for: Obstacles to successful treatment of hepatitis C in uninsured patients from a minority population
Source: J Transl Med. 2018 Jun 28;16:178. doi: 10.1186/s12967-018-1555-y (PMC6027772; doi:10.1186/s12967-018-1555-y)

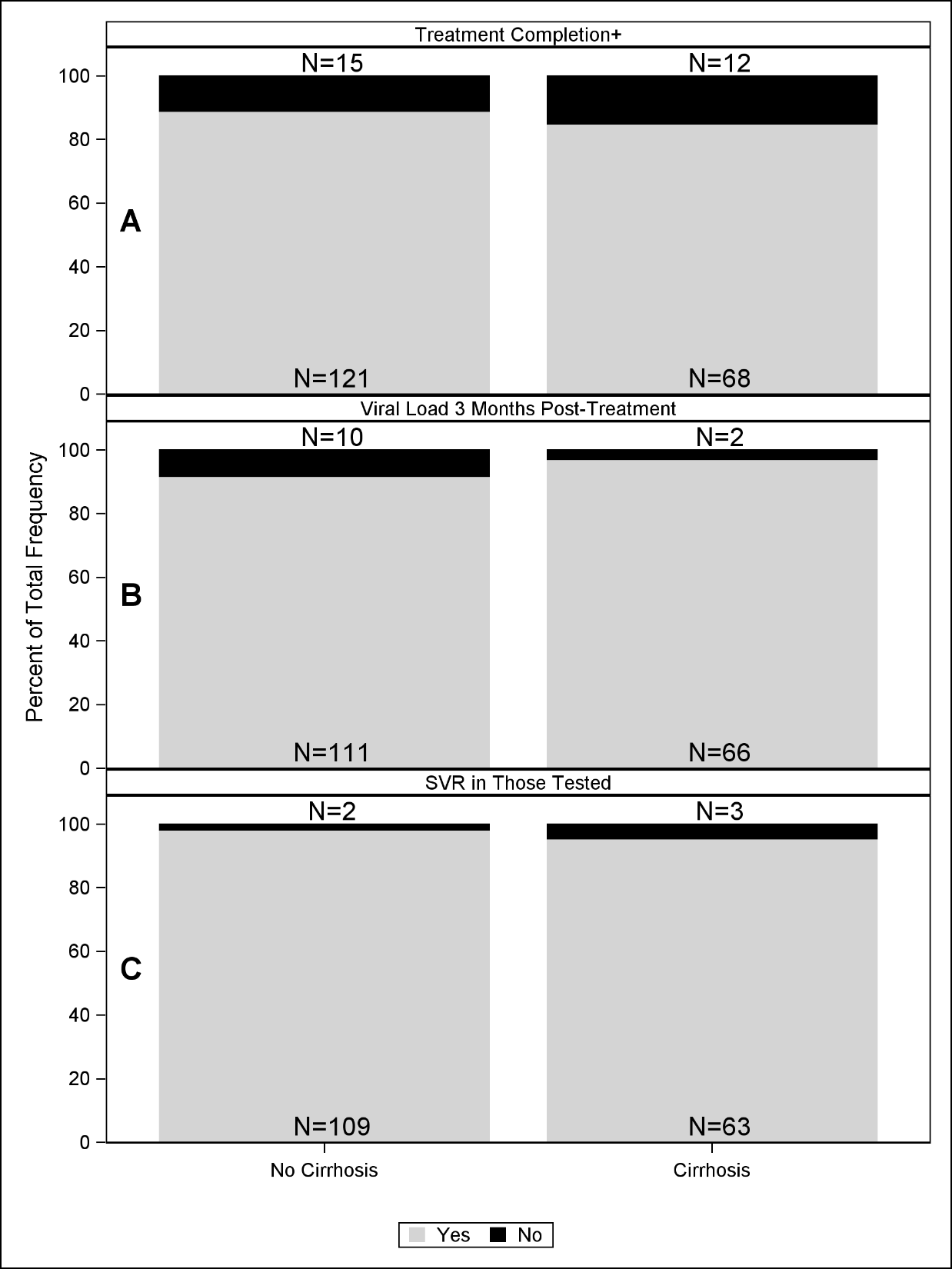


**Additional file 1: Figure S1**.

Supplement: Supplementary file 1 — Additional file 1: Figure S1. Attainment of specific milestones in the HCV treatment cascade of cirrhotic and non-cirrhotic patients. A. In patients who initiated treatment (N = 216). B. In patients who completed treatment (N = 189). C. In patients who obtained a 12-week post treatment viral load (N = 177). † Completed treatment per guidelines (based on patient report). [file 12967_2018_1555_MOESM1_ESM.docx]
