# Supplementary material for: Obstacles to successful treatment of hepatitis C in uninsured patients from a minority population
Source: J Transl Med. 2018 Jun 28;16:178. doi: 10.1186/s12967-018-1555-y (PMC6027772; doi:10.1186/s12967-018-1555-y)
Supplement: Supplementary file 2 — Additional file 2: Table S1. Univariable and multivariable logistic regression for association with treatment completion. [file 12967_2018_1555_MOESM2_ESM.docx]

**Additional file 2: Table S1**. Univariate and multivariable logistic regression for association with treatment completion.

|  | Univariate Logistic Regression | | Multivariable Logistic Regression | |
| --- | --- | --- | --- | --- |
|  | Odds Ratio (95% CI) | p-value | Adjusted Odds Ratio (95% CI) | p-value |
| Age at Treatment, Years | 0.94 (0.904,0.976) | **0.0015** | 0.955 (0.91,1.003) | 0.0636 |
| Sex |  |  |  |  |
| Female vs Male (ref) | 0.312 (0.126,0.771) | **0.0117** | 0.366 (0.127,1.056) | 0.0631 |
| Race |  |  |  |  |
| Non-White vs White (ref) | 0.831 (0.333,2.077) | 0.6924 | - | - |
| Black vs White (ref) | 0.895 (0.357,2.243) | 0.8132 | - | - |
| Asian/Other vs White (ref) | NE | NE | - | - |
| Ethnicity |  |  |  |  |
| Hispanic vs Non-Hispanic | 0.562 (0.249,1.267) | 0.1647 | - | - |
| Race/Ethnicity |  |  |  |  |
| Hispanic vs  Non-Hispanic White (ref) | 0.405 (0.152,1.082) | 0.0713 | 0.297 (0.089,0.994) | 0.0489 |
| Non-Hispanic Black vs  Non-Hispanic White (ref) | 0.583 (0.191,1.778) | 0.3432 | 0.764 (0.202,2.895) | 0.6917 |
| Asian/Other vs  Non-Hispanic White (ref) | NE | NE | NE | NE |
| Compliance |  |  |  |  |
| No vs Yes (ref) | 9.455 (3.906,22.883) | **<.0001** | 11.865 (4.073,34.569) | **<.0001** |
| Complications |  |  |  |  |
| Yes vs No (ref) | 0.727 (0.323,1.636) | 0.4415 | 1.118 (0.397,3.149) | 0.8331 |
| Pre-Treatment Viral Load, IU/mL |  | 0.4152 |  | 0.5225 |
| 1,000,000 unit increase | 0.957 (0.861,1.064) |  | 0.961 (0.850,1.086) |  |
| Insurance Status |  |  |  |  |
| No vs Yes (ref) | 1.473 (0.564,3.845) | 0.4287 | 1.267 (0.389,4.134) | 0.6944 |
| Genotype |  |  |  |  |
| non-1 vs 1 | 1.214 (0.457,3.227) | 0.6969 | 0.915 (0.276,3.031) | 0.8843 |
| 2 vs 1 | 0.81 (0.175,3.74) | 0.7867 | - | - |
| 3 vs 1 | 2.649 (0.773,9.081) | 0.1211 | - | - |
| 4 vs 1 | NE | NE | - | - |
| mixed vs 1 | NE | NE | - | - |
| Cirrhosis |  |  |  |  |
| Yes vs No (ref) | 1.424 (0.63,3.217) | 0.3957 | 2.949 (0.992,8.77) | 0.0518 |

**Additional file 2: Table S1**. Univariate and multivariable logistic regression for association with treatment completion per guidelines (based on patient report) (N=216). Multivariable logistic regression included all variables. CI = confidence interval.
