# Supplementary material for: Obstacles to successful treatment of hepatitis C in uninsured patients from a minority population
Source: J Transl Med. 2018 Jun 28;16:178. doi: 10.1186/s12967-018-1555-y (PMC6027772; doi:10.1186/s12967-018-1555-y)
Supplement: Supplementary file 3 — Additional file 3: Table S2. Univariable and multivariable logistic regression for association with being tested for SVR. [file 12967_2018_1555_MOESM3_ESM.docx]

**Additional file 3: Table S2**. Univariate and multivariable logistic regression for association with being tested for SVR.

|  | Univariate Logistic Regression | | Multivariable Logistic Regression | |
| --- | --- | --- | --- | --- |
|  | Odds Ratio (95% CI) | p-value | Adjusted Odds Ratio (95% CI) | p-value |
| Age at Treatment, Years | 0.992 (0.929,1.058) | 0.7985 | 1.033 (0.925,1.154) | 0.5672 |
| Sex |  |  |  |  |
| Female vs Male (ref) | 0.883 (0.274,2.843) | 0.8348 | 1.004 (0.154,6.558) | 0.9971 |
| Race |  |  |  |  |
| Non-White vs White (ref) | 0.78 (0.203,2.995) | 0.7173 | - | - |
| Black vs White (ref) | 0.844 (0.219,3.247) | 0.8046 | - | - |
| Asian/Other vs White (ref) | NE | NE | - | - |
| Ethnicity |  |  |  |  |
| Hispanic vs Non-Hispanic | 0.686 (0.213,2.21) | 0.5275 | - | - |
| Race/Ethnicity |  |  |  |  |
| Hispanic vs  Non-Hispanic White (ref) | 0.371 (0.098,1.413) | 0.1462 | <0.001 (<0.001,>999.999) | 0.901 |
| Non-Hispanic Black vs  Non-Hispanic White (ref) | 0.302 (0.052,1.768) | 0.1843 | <0.001 (<0.001,>999.999) | 0.8886 |
| Asian/Other vs  Non-Hispanic White (ref) | NE | NE | - | - |
| Compliance |  |  |  |  |
| No vs Yes (ref) | NE | NE | NE | NE |
| Complications |  |  |  |  |
| Yes vs No (ref) | 0.903 (0.28,2.908) | 0.8645 | 0.497 (0.066,3.744) | 0.4973 |
| Pre-Treatment Viral Load, IU/mL |  | 1 |  | 0.9851 |
| 1,000,000 unit increase | 1.000 (0.958,1.044) |  | 1.002 (0.845,1.188) |  |
| Insurance Status |  |  |  |  |
| No vs Yes (ref) | 0.832 (0.24,2.884) | 0.7718 | 0.133 (0.008,2.121) | 0.1532 |
| Genotype |  |  |  |  |
| non-1 vs 1 | 1.455 (0.373,5.669) | 0.5893 | 2.365 (0.237,23.58) | 0.4631 |
| 2 vs 1 | NE | NE | - | - |
| 3 vs 1 | 3.556 (0.667,18.957) | 0.1374 | - | - |
| 4 vs 1 | 3.2 (0.337,30.366) | 0.311 | - | - |
| mixed vs 1 | NE | NE | - | - |
| Cirrhosis |  |  |  |  |
| Yes vs No (ref) | 0.336 (0.072,1.582) | 0.1679 | 0.154 (0.007,3.365) | 0.2342 |

**Additional file 3: Table S2**. Univariate and multivariable logistic regression, in patients who completed treatment, for association with obtaining a 12-week post treatment viral load (N=189). Multivariable logistic regression included all variables. CI = confidence interval.
